# Customised, individually made total knee arthroplasty shows promising 1-year clinical and patient reported outcomes

**DOI:** 10.1007/s00402-021-04045-1

**Published:** 2021-07-16

**Authors:** Céline S. Moret, Michael T. Hirschmann, Nicole Vogel, Markus P. Arnold

**Affiliations:** 1 Department of Orthopaedic Surgery and Traumatology, University of Basel, Kantonsspital Baselland, 4101 Bruderholz, Switzerland; 2 Practice Leonardo, Hirslanden Klinik Birshof, Reinacherstrasse 28, 4142 Münchenstein, Switzerland

**Keywords:** Total knee arthroplasty, Customised individually made, patient-specific implants, Knee Society Score, Patient reported outcome measures, Satisfaction

## Abstract

**Introduction:**

Customised individually made (CIM) implants for total knee arthroplasty (TKA) were introduced about 10 years ago. These implants aim to reduce the risk of prosthesis-related issues resulting from anthropometric differences between different knees.

The purpose of this study was to analyse the short-term clinical outcome and patient reported outcome measures (PROMs) of a specific CIM implant, the ORIGIN^®^ knee replacement system (Symbios, Yverdon-les-Bains, Switzerland), which was introduced in 2018.

**Materials and methods:**

This is a prospective cohort study of patients undergoing primary posterior-stabilised (PS) CIM TKA using the specific ORIGIN^®^ knee replacement system, (Symbios, Yverdon-les-Bains, Switzerland). TKAs were performed from February 2019 to October 2020. Data was collected preoperatively and postoperatively at 4 and 12 months. Outcome measures included the objective part of the Knee Society Score (KSS) with the range of motion (ROM) and the following PROMs: the Knee injury and Osteoarthritis Outcome Score (KOOS), the Forgotten Joint Score (FJS-12), the EuroQol, five dimensions, three levels (EQ-5D-3L) with the EuroQol visual analogue scale (EQ-VAS) and patient satisfaction. Differences in pre- to preoperative data were assessed with paired sample *t* tests. A *p* value < 0.05 was considered significant.

**Results:**

Twenty-five CIM TKA (20 patients, 8 female) were included. The mean age at surgery was 66 years (SD, 6.9). At 4 and 12 months, significant improvements in the KSS (*p* < 0.001), the ROM (*p* < 0.001), all KOOS subscales (*p* < 0.001), the FJS (*p* < 0.001) and the EQ-5D-3L (*p* < 0.026) were found. Satisfaction rate was 91% and 88% at 4 and 12 months, respectively. Intraoperative complications did not occur and no revision surgeries were undertaken.

**Conclusions:**

The present study demonstrated significant improvements in the KSS and specific PROMs 1 year after CIM TKA. This study suggests that CIM TKA is a safe and suitable option, which can yield good clinical outcome and PROMs at least during short-term follow-up.

## Introduction

About 20% of patients are unsatisfied after primary total knee arthroplasty (TKA). Patients report persisting pain, instability, stiffness or a persistent or recurrent effusion requiring a subsequent revision [1–8]. In particular, aseptic loosening, instability and patellofemoral disorders, which are responsible for about 40% of all revision causes, are known to be affected by TKA component size or positioning [6, 7]. These issues might theoretically be reduced by a more individualised, patient-specific approach. Customised individually made (CIM) TKA requires patient-specific implants and instrumentation to better fit the individual anthropometric knee joint characteristics.

Hirschmann et al. [8–10] have shown a high variability of the medial femoral mechanical angle (mFMA) and the medial tibial mechanical angle (mTMA) within the three classical limb phenotypes in a non-osteoarthritic Caucasian population. They recommended that the overall coronal lower limb alignment should not only be classified in neutral, varus or valgus on the basis of the hip–knee–ankle angle (HKA), but that femoral and tibial joint lines should be considered as well. Furthermore, several studies have demonstrated that among different ethnic groups different mediolateral-to-anteroposterior ratio of tibia and femur exist [11–13].

Conventional, off-the-shelf (OTS) implants are based on anthropometric measurements of a defined standard, however, mostly Caucasian population [11]. Although multiple models and sizes of OTS implants exist, it can be challenging to find the most adapted for the patient’s knee morphology. The surgeons’ experience with different implant models or the availability in a specific hospital can also play a role in the choice of the implant. Thus, quite recently developed CIM implants are specifically adapted to the individual knee morphology, especially in patients who present less conventional anthropometric characteristics. Hence, the aim of this study was to analyse the clinical outcome and specifically patient reported outcome measures (PROMs) after TKA with a specific CIM implant. It was hypothesised that CIM TKA shows good short-term clinical and patient reported outcomes.

## Materials and methods

### Study design and population

This study was approved by the local ethics committee (reference: 2016-01777) and written informed consent was obtained from all patients willing to participate.

This is a single-site, prospective cohort study including patients undergoing primary posterior-stabilised (PS) CIM TKA using the ORIGIN^®^ knee replacement system (Symbios, Yverdon-les-Bains, Switzerland). Routinely, all patients scheduled for a TKA were asked to complete PROMs before and after the surgery. The CIM TKAs were performed between February 2019 and October 2020 in a private hospital by one experienced board-certified surgeon specialised in knee surgery (MPA).

The decision whether to implant the ConforMIS^®^ or the ORIGIN^®^ CIM knee replacement system depended on the integrity of the posterior cruciate ligament (PCL) and on the preoperative knee flexion. In patients with intact PCL the cruciate-retaining (CR) version of the ConforMIS^®^ knee replacement system was opted for whereas patients with either preoperative or expected intraoperative PCL insufficiency or passive flexion of less than 110° underwent CIM TKA with the ORIGIN^®^ PS knee replacement system. Inclusion criteria for the ORIGIN^®^ CIM TKA were primary TKA, non-inflammatory degenerative or inflammatory disease, less than 10° of recurvatum, a coronal varus or valgus HKA deviation of less than 10°, a pre-existing PCL lesion or the presence of large posterior osteophytes, which would lead to a PCL insufficiency after their removal, an absence of collateral ligament distention and/or excessive extra-articular deformation. Patients with insufficient knowledge of German, English, French or Italian were excluded.

Between February 2019 and October 2020, 35 CIM TKAs (29 patients, 12 female) were performed. Of those, 25 CIM TKAs (20 patients, 8 female) had available PROMs and were thus included in this study. Five patients had a bilateral CIM TKA and three had a contralateral TKA other than CIM TKA. The mean age at surgery was 66 years (SD, 6.9). Patient demographics are described in Table 1.

**Table 1:** Patient demographics

Variables at surgery		CIM TKA (*n* = 25)
Age, mean (SD)		66 years (6.9)
Body mass index, mean (SD)		28.1 kg/m^2^ (5.5)
Sex, *n* (%)	Male Female	16 (64) 9 (36)
Side, *n* (%)	Right Left	12 (48) 13 (52)
Kellgren–Lawrence score, *n* (%)	3 4	2 (8) 23 (92)
Alignment, *n* (%)	Neutral Varus Valgus	4 (16) 17 (68) 4 (16)

*CIM TKA* customised, individually made total knee arthroplasty,* SD* standard deviation

### Implant design, production process and surgical technique

**Fig. 1:**
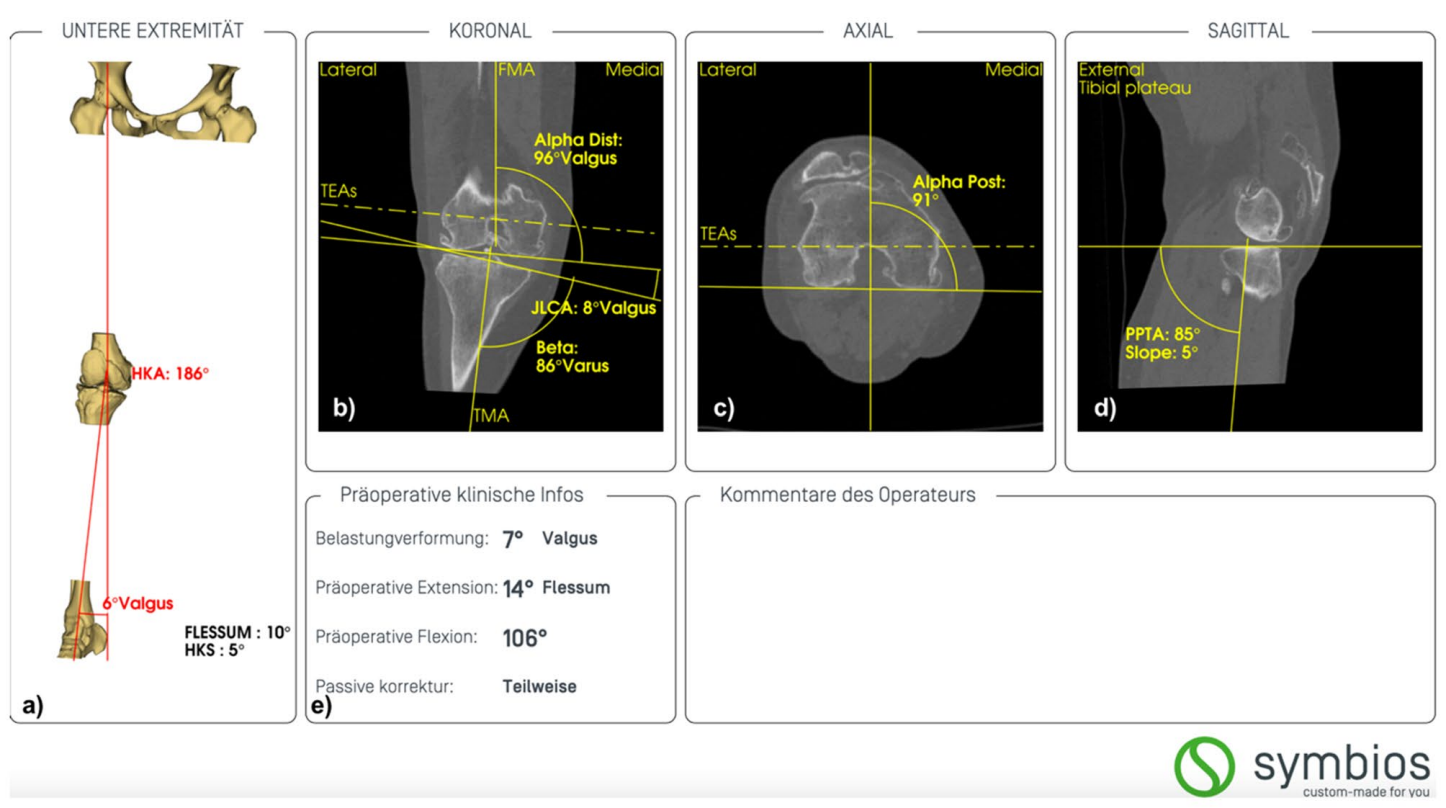
Example of preoperative analysis in a female patient with valgus osteoarthritis of her right knee (KNEE-PLAN^®^, Symbios). **a** Determination of the limb alignment with the hip-kneeankle angle (HKA) and the hip-knee-shaft angle (HKS) in the coronal plane. **b** Determination of the mechanical medial distal femoral angle (Alpha Dist), the mechanical medial proximal tibial angle (Beta), and the joint line convergence angle (JLCA) in the coronal plane. The femoral mechanical angle (FMA), the tibial mechanical angle (TMA) and the surgical transepicondylar axis (TEAs) are marked as reference. **c** Determination of the rotational alignment (Alpha post) of the distal femur in the axial plane. In this case, the posterior condylar angle is 1°. **d** Determination of the posterior proximal tibial angle (PPTA) in the sagittal plane. In this case, the posterior slope is 5°. **e** Clinical information provided by the surgeon

The implant is based on the preoperative computed tomography of the osteoarthritic knee, according to the Imperial Knee Protocol, and on its subsequent three-dimensional (3D) reconstruction [14]. The alignment and morphology of the knee are analysed measuring different angles and axis using a specialised planning software by the manufacturer’s engineer (Knee-Plan^®^, Symbios, Yverdon-les-Bains, Switzerland) (Fig. 1).

After assessment of cartilage wear, subchondral bone loss and location of osteophytes of the femur and tibia, the engineer determines in a best-fit scenario the individual femoral J-curves. The ORIGIN^®^ realignment strategy is a personalised postoperative alignment according to the restricted phenotype alignment protocol, which aims to reproduce the individual coronal knee phenotype in the limits of a safe target zone [15]. The tibial baseplate is asymmetric to fit the native tibial plateau and the angle of the tibia keel is adapted to the metaphysis [16]. The design and fitting of the components are reproduced with a 3D software (SolidWorks^®^ software, Dassault Systèmes, Vélizy-Villacoublay, France) [16]. This allows a reproduction of asymmetries, an optimal coverage of the resected bone surface by the implant and an anteroposterior (AP) to mediolateral (ML) ratio adapted to each patient. In addition, ligament balancing is improved due to the reproduction of the patient’s pre-osteoarthritic anatomy [16]. Thus, resection laxity due to asymmetric bone cuts can be avoided [16].

**Fig. 2:**
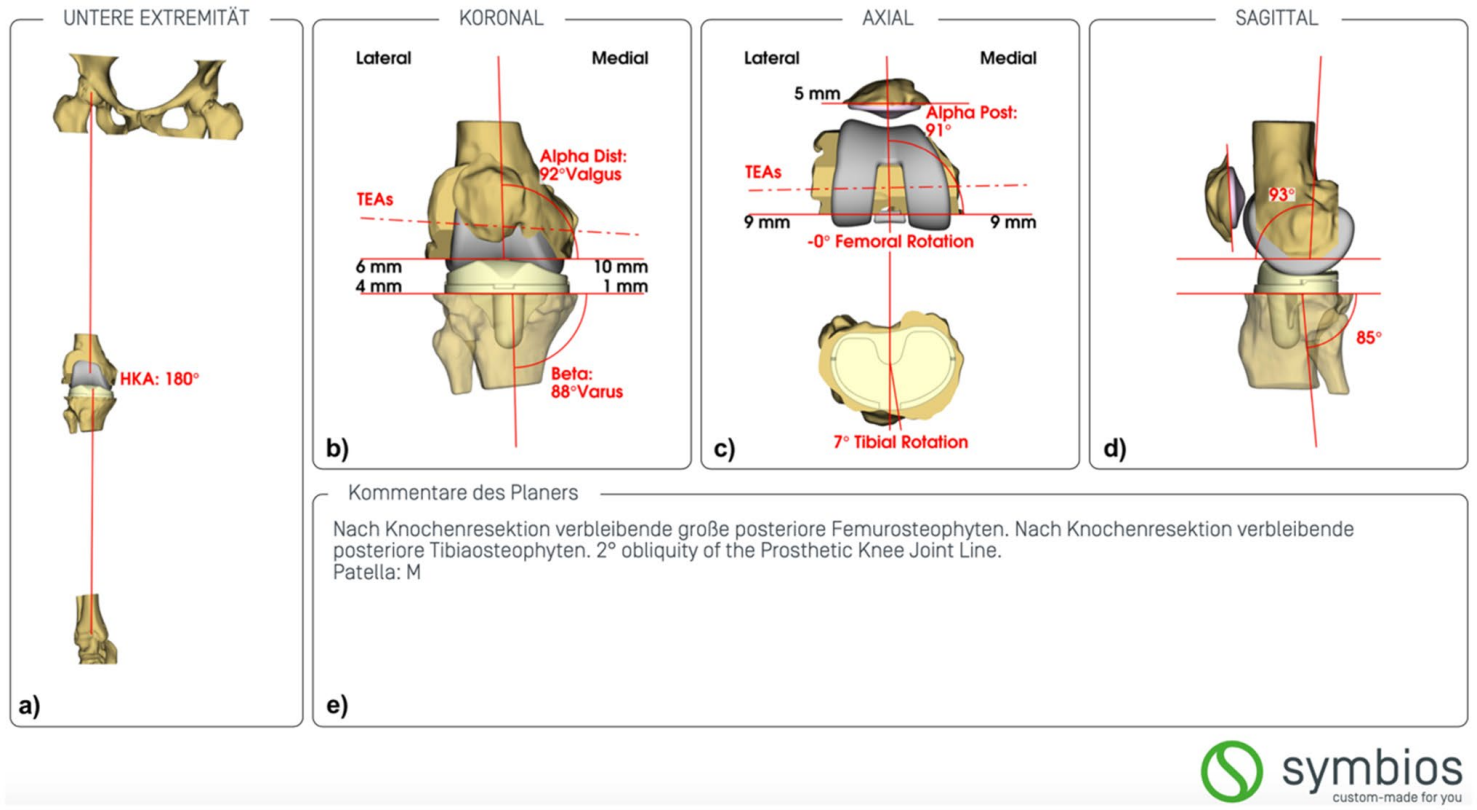
Example of the planning summary suggested for the same patient as seen in Fig. 1. **a** Restoration of the limb alignment to a hip-knee ankle angle (HKA) of 180° in this case. **b** Restoration to a mechanical medial distal femoral angle (Alpha Dist) and mechanical medial proximal tibial angle (Beta) of 92° and 88° in this case, respectively. **c** Axial rotational alignment of the femoral component and tibial component. **d** Sagittal alignment of the femoral and tibial component. **e** Comments from the engineer

After validation of the planning summary (Fig. 2) by the surgeon, the production of the CIM knee system is undertaken. It comprises a customised PS tibial and femoral component, with or without patellar component, and individually printed custom-cutting-blocks for patient-specific instrumentation. Anteroposterior stability relies on the ultra-congruent polyethylene for the first 60° of flexion, then the post-cam system engages [16]. The prosthesis has a chrome-cobalt-molybdenum (CrCoMo) femoral and a titanium alloy (Ti6Al4V) tibial component with a snap fit, fixed bearing, ultra-congruent, ultrahigh molecular weight polyethylene (UHMWPE) insert. Both components are cemented.

The CIM TKA was usually performed through a medial parapatellar approach with individually printed custom-cutting-blocks and custom trials. In tight valgus knees, a lateral approach with osteotomy of the tibial tuberosity was preferred [17]. For optimal positioning and orientation of the cutting blocks, the surgeon aimed for a best-fit to the bone that ensured optimal stability of the four cutting block zones resting on the bone as presented on the provided three-dimensional model of the femur and tibia. The varus or valgus alignment of the tibial cutting block was assessed with an extramedullary reference rod which helped to determine if a slight correction before resection was required.

All patients received the same standard postoperative follow-up and physical therapy.

### Clinical outcome and PROMs

The following parameters were assessed preoperatively and postoperatively after four and 12 months during routine follow-up consultations according to the publication by Vogel et al. [18]. The surgeon assessed the objective part of the Knee Society Score (KSS), which measures pain, range of motion (ROM), stability and alignment. Its score varies between 0 and 100 [19].

Following PROMs were completed: (1) the Knee injury and Osteoarthritis Outcome Score (KOOS), (2) the Forgotten Joint Score (FJS-12) and (3) the EuroQol five dimensions, three levels (EQ-5D-3L). The KOOS consists of five subscales and captures pain, symptoms, activities of daily living (ADL), sport and recreational activities and knee-related quality of life (QoL). Each subscale has a score ranging from 0 to 100 [20]. The Forgotten Joint Score assesses joint awareness with a total score from 0 to 100 points [21]. The EQ-5D-3L evaluates health-related quality of life with an index score ranging from 0 to 1 and a and visual analogue scale (EQ-VAS) ranging from 0 to 100 points [22].

In the KSS and PROMs, a higher total score indicates a better outcome.

Moreover, patients’ satisfaction with the surgery was evaluated on a five-point Likert scale ranging from “very satisfied” to “very unsatisfied”. Patient’s willingness to undergo CIM TKA again (yes/no) was also assessed during follow-up after 12 months.

Intraoperative complications and revision surgeries were also recorded.

All study data were collected and managed with the Research Electronic Data Capture (REDCap).

### Statistical method

Statistical analysis was performed with IBM SPSS Statistics for Windows version 27 (IBM Corp., Armonk, NY, US). Descriptive statistics are presented with mean and standard deviation (SD) for continuous variables, frequency counts and percentages for categorical variables. Normal distribution was confirmed with the Kolmogorov–Smirnov test and the paired sample t test was applied to determine pre- to postoperative differences of continuous variables. Results are presented with a 95% confidence interval (CI) and a *p* value of < 0.05 was considered significant.

## Results

The data of 25 and 16 CIM TKA were analysed at 4 and 12 months, respectively. There were no losses to follow-up. A neutral and a varus limb alignment were attained in 24 and 1 CIM TKA, respectively. There were significant pre- to postoperative improvements in KSS (*p* < 0.001), ROM (*p* < 0.001), all KOOS subscales (*p* < 0.001), FJS (*p* < 0.001) and EQ-5D-3L (*p* < 0.026) at both follow-up consultations. The EQ-VAS improved nonsignificantly. Detailed results can be seen in Table 2.

**Table 2:** Clinical outcome and PROMs at different time-points

Measurement	CIM TKA				
	Preoperative	4-month follow-up		12-month follow-up	
	*n* = 25 Mean (SD)	*n* = 25 Mean (SD)	Difference to preoperative *p* value (95% CI)	*n* = 16 Mean (SD)	Difference to preoperative *p* value (95% CI)
oKSS	46 (13.9)	90 (6.1)	< 0.001 (37.2–50.8)	94 (5.8)	< 0.001 (38.3–56.9)
ROM	109 (16.7)	125 (5.7)	< 0.001 (7.8–19.6)	129 (5.3)	< 0.001 (11.9–28.1)
KOOS symptoms	44 (12.9)	68 (13.7)	< 0.001 (13.7–33. 7)	80 (8.5)	< 0.001 (34.1–47.9)
KOOS pain	50 (14.3)	75 (12.6)	< 0.001 (16.4–33.5)	86 (11.4)	< 0.001 (25.9–47.5)
KOOS ADL	60 (16.9)	79 (14.7)	< 0.001 (9.3–29.2)	87 (10.5)	< 0.001 (16.8–34.8)
KOOS sports	26 (18.8)	56 (25.1)	< 0.001 (16.3–41.0)	64 (30.8)	< 0.001 (26.9–58.5)
KOOS QoL	33 (15.9)	58 (25.1)	< 0.001 (14.2–35.8)	70 (20.3)	< 0.001 (25.0–51.3)
FJS-12	22 (14.7)	52 (23.7)	< 0.001 (19.1–42.3)	73 (19.7)	< 0.001 (40.7–63.5)
EQ-5D-3L	0.707 (0.16)	0.824 (0.16)	< 0.026 (0.155–0.221)	0.914 (0.11)	0.001 (0.105–0.308)
EQ-VAS	75 (16.9)	78 (17.6)	0.548 (− 7.4 to 13.5)	84 (10.5)	0.164 (− 3.7 to 20.1)

*oKSS* objective part of the Knee Society Score,* ROM* range of motion, *KOOS* Knee injury and Osteoarthritis Outcome Score,* ADL* activities of daily living, *QoL* quality of life, *FJS-12* Forgotten Knee Joint Score,* EQ-5D-3L* EuroQol five dimensions three levels, *VAS* visual analogue scale, *SD* standard deviation, *CI* confidence interval

At 4 and 12 months, 91% and 88% of patients reported to be very satisfied or satisfied with their knee implant, respectively, whereas the remaining patients were neither satisfied nor dissatisfied. After 12 months, all patients would undergo the same surgery again.

The patient-specific instrumentation (PSI) fitted seamlessly to the bony morphology. The pins were stable and no repositioning was necessary. No other intraoperative complications or revision surgeries during the follow-up period occurred.

## Discussion

The most important finding of the present study is the safety and reliability of the ORIGIN^®^ CIM knee replacement system, which shows good short-term clinical and patient reported outcomes.

There are currently no available publications assessing non-radiological clinical and patient reported outcomes after CIM TKAs with the ORIGIN^®^ knee replacement system. Hence, a comparison can only be undertaken with results of the CIM ConforMIS^®^ knee replacement system. The present study demonstrated, as expected, a significant pre- to postoperative improvement in the objective part of the KSS at 4 and 12 months. This improvement is comparable to the difference of 38.3 points (SD, 14.4) found at 3 months by Wheatley et al. [23]. The same author, however, detected no significant differences in the objective part of the KSS between the CIM and the OTS TKA groups [23]. Reimann et al. [24] only found a significant increase in the function score (subjective part) of the KSS, thus leading to a significantly better entire KSS, which the authors attribute to a younger mean age of 65 years in their CIM TKA cohort, whereas White and Ranawat [25], who compared 74 patient knees 2 years after undergoing TKA with different implants (CIM TKA:21/OTS TKA:11/PS OTS TKA:42) even found worse scores in the objective and subjective parts of the KSS as well as lower satisfaction rates in patients with CIM implants.

Concerning the ROM, Schwarzkopf et al. [26], analysing 621 TKAs (CIM implants:307/OTS implants:314), measured a decrease of 3.44° 1 year postoperatively compared to preoperatively in the CIM TKA groups; a result, the authors considered without any clinical significance due to their non-identical ROM measurement protocol. Moreover, comparison of the ROM between the two groups at a minimum of 1 year, did not show a significant difference [24–26].

Almost one-third of patients required manipulation under anaesthesia (MUA) to improve the ROM to a mean of 115° at 2 years in the CIM TKA cohort of White et Ranawat [25]. These findings are not in line with the present study, showing no MUA or revision and a mean ROM of 125° and 129° at 4 and 12 months, respectively.

Potential stiffness leading to a reduced ROM might be explained with the limited options a surgeon has at hand intraoperatively in terms of resection depth. As only two inlay heights (0 and + 2 mm) are available, some surgeons tend to underresect and thus render the knee rather tight in extension and flexion. This is not a problem related to the CIM implant itself, but to the PSI. There is well-established evidence about the accuracy and limitations of PSI with regards to the implant orientation [27–30]. When assessing the coronal alignment of the tibial component, Zahn et al. [31] observed more outliers from the neutral mechanical medial proximal tibial angle in the PSI group than in groups comprising extramedullary or intramedullary implant positioning technique or computer-navigated implantation. From experience, ConforMIS^®^ PSI for the tibial component have a tendency to tilt downward when fixed to the tibia, hence recreating less posterior slope than planned. This occurs less frequently with the ORIGIN^®^ PSI, as the ORIGIN^®^ tibial guide uses different and more reliable reference points.

Concerning PROMs, Reimann et al. [24] found no significant differences between the CIM and OTS TKA group 2–3 years after surgery in all KOOS subscales. This shows that complex everyday activities (e.g. getting in the bath, gardening or getting out of the car) still remain challenging after a TKA independently of the utilization of CIM or OTS implants [24]. In the present study, the lowest mean score of 64 points was attained in the subscale sports. This result builds on the data from Reimann et al. [24] who found also lower mean scores (< 60 points) in the same subscale for both CIM TKA and OTS TKA groups, explained by the apprehension or refusal of many patients to run, jump or kneel after TKA. The present study showed a significant mean pre- to postoperative improvement in the FJS-12 with a mean score of 73 points (SD 19.7) at 12 months. This result is within the range of 67.6 (SD 27.8) to 82 (range, 70–94) found for OTS implants in different publications [32–34]. Similarly, Wheatley et al. [23], revealed no significant difference in the FJS-12 between patients who underwent CIM or OTS TKAs at a mean of 2 years postoperatively.

Patient satisfaction after CIM TKA was higher at 4 months (91% vs 83%) and slightly lower at 12 months (88% vs 91%) compared to a publication about OTS TKA [35]. This slightly worse result at 1 year might be due to the fact that potentially satisfied and very satisfied patients have not yet attended the 12-month follow-up. Nevertheless, satisfaction rate of the included patients is well above patient satisfaction of approximately 80% recorded in multiple other studies [1–8]. Despite the lack of long-term evidence that CIM TKA directly improves clinical and patient outcomes, Reimann et al. [24], who compared patient satisfaction of 84 CIM TKAs with 57 OTS TKAs 2–3 years after surgery, found that global satisfaction was significantly better in the CIM TKA group. This result might be partially attributed to a placebo effect since patients are aware that they have received the latest generation of TKA implants.

Recent articles have shown that anteroposterior to mediolateral femoral and tibial ratios differ between ethnic groups and that component overhang might lead to knee pain [11–13, 36]. CIM implants provide better cortical bone coverage and thus reduce the risk of overhang and under-coverage [37]. Indeed, Klasan et al. [38] demonstrated that overhang of the tibial component decreased the clinical outcome in terms of KOOS by the same margin as loss of 16% of coverage. Furthermore, optimal bone coverage could lower the risk of bleeding from resected bone surfaces, reducing postoperative knee swelling as well as potentially allowing a better ROM and physical therapy participation postoperatively [26, 39, 40].

Whether a CIM TKA is associated with less blood transfusions, less intraoperative blood loss or a lower drop in haemoglobin postoperatively compared to OTS TKA remains controversial [26, 40, 41].

Anterior knee pain is a well-known problem after TKAs [42]. With CIM implants, the trochlea is designed to match the shape of the native patella and to maintain its native alignment, thus reducing patellar maltracking and the risk for secondary patellar resurfacing [16, 43].

The great variation in knee morphology among patients was seen in publications by Hirschmann et al. [8–10] assessing the association of limb alignment with the tibial and femoral joint line of 308 non-osteoarthritic patient knees. The authors determined eight phenotypes (i.e. 20% of all phenotypes found), which represent the coronal knee morphology of nearly 75% of the population. In contrast, the mechanical, anatomical and restricted kinematic alignment matched the phenotypes of only 5%, 20% and 51% of the population, respectively [10]. Thus, with the ORIGIN^®^ alignment, which is based on the restricted phenotype alignment, the patient specific-knee morphology and limb alignment is better reproduced. Nevertheless, the comparison between CIM and OTS implants demonstrated that the former involved more physiological knee kinematics with greater weight bearing knee flexion and more posterior femoral rollback as well as greater axial rotation [44–46].

It is important to note that a measured resection technique bears the risk of underestimating an unequal and asymmetric laxity of the knee joint. This might lead to a less well-balanced knee. Therefore, a more constrained TKA should be carefully considered for gross deformities. Similarly, a further aspect is the risk for the surgeon to rely heavily on the engineer’s preoperative rather than on the intraoperative assessment [26].

The most relevant limitation of this study is the missing control group receiving standard OTS implants, thus these results can only be compared with the findings of other studies, which checked CIM versus OTS implants. The available literature, however, used different CIM implants and outcome measures. Second, the number of included patients is quite small and the follow-up is limited to 1 year, since the ORIGIN^®^ knee replacement system is only available since 2018. There is evidence showing an average time to revision below 1 year among patients with early knee implant failures [47]. Thus, despite the short follow-up time, preliminary results as presented in this study are of relevance. Third, CIM TKA are mostly performed in a private hospital setting and patient selection might be biased as most patients had an additional private insurance, which reflects a higher socioeconomic status. It is well known that these patients show increased satisfaction and better function. The demographics of the included patients do not fully overlap with the average patients undergoing TKA. Indeed, the former are younger and have a lower BMI, while the male gender is overrepresented [48–50].

## Conclusions

This study demonstrated significant improvements in the KSS and specific PROMs 1 year after CIM TKA. With a satisfaction rate of 88% and no postoperative complications, this study suggests that TKA performed with a CIM implant is a safe and suitable option, which can yield good outcomes in terms of clinical outcome and PROMs at least in the short-term. Further studies should assess outcome scores and PROMs between CIM TKA and OTS TKA with different realignment strategies.
